# Medical Communications of the Massachusetts Society 1885

**Published:** 1886-04

**Authors:** 


### Book Reviews.

MEDICAL COMMUNICATIONS OF THE MASSACHUSETTS MEDICAL SOCIETY. VOL. XIII. NO. IV. 1885.

The Massachusetts Medical Society is one of the oldest and most respectable professional bodies in this country, and its communications are always of a high order of excellence. The present volume forms no exception in this respect.

The volume opens with the annual address of the President, Dr. F. K. Paddock, upon "Antiseptic Surgery," giving what are claimed to be the accepted views upon the subject. As a *resume* of the ideas of the most extreme advocates of Listerism, the article might be said to cover the whole ground. But as an exposition of the present aspect of the question of antisepsis, it is far from complete, since it ignores the experience of such eminent authorities as Hamilton, Emmet, Tait, Keith, Savage, Bryant, Bantock and many others who have discarded Listerism after an impartial trial. In view of the results obtained by Lawson Tait in abdominal surgery, without the use of antiseptics, the statement that "the astonishing success attained in this particular operation (ovariotomy) is unquestionably the result of the faithful execution of the details involved in the antiseptic method" is not warranted by facts.

A very readable article by Dr. John Homans deals with the interesting but somewhat hackneyed subject of "The Influence of Ovariotomy on Surgery." The author states that he has performed 270 laparotomies, of which number 200 were ovariotomies for the removal of cystic ovaries. But he fails to give his percentage of mortality, thereby omitting the chief point which would render his experience valuable as a contribution to the statistics of the subject.

Perhaps the most important communication of this volume, in that it gives valuable results of original investigation, is the article, by Dr. Charles F. Withington, upon "Consanguineous Marriages: their Effect upon Offspring." The author has tabulated 108 cases of consanguineous marriages which have come under his own observation. From these marriages 413 children resulted, of which number 312, or seventy-eight per cent., "appeared to be free from any congenital defect or disease, and had an average degree of intelligence and bodily well-being." The conclusions deduced by the author are contrary to those commonly accepted and are summed up as follows: "The total results are not such as to show any special or conspicuous deterioration peculiar to the children of relations."

An article, by Dr. William L. Richardson, upon "The Diagnosis and Treatment of Posterior Positions of the Occiput" is deserving of mention. It emphasizes the importance of an early diagnosis, not only of the presentation, but of the position of the fœtal head, and advocates abdominal palpation as an easy means of making such a diagnosis. The author's views will be fully corroborated by every practitioner who has educated himself to this method of examination. But the *tactus eruditus* is a very essential requisite to the successful employment of this method, and the results obtained by the novice will be far from satisfactory. In the hands of the expert, however, its value cannot be over-estimated.

Other articles in this volume are "Pregnancy and Labor, Complicated with Fibroids," by Dr. James R. Chadwick; "The Pathogenesis of Certain Affections of the Skin," by Dr. George H. Tilden; "Some of the Mental Aspects of Nervous Disease,"

by Dr. Henry R. Stedman; "Cremation in its Sanitary Aspects," by Dr. John O. Marble, and "How a Lesion of the Brain Results in Sensory Aphasia," by Dr. Morton Prince. V. O. H.

